# Lower First Permanent Molar with an Additional Root Diagnosis and Management

**DOI:** 10.1155/2019/8403140

**Published:** 2019-12-23

**Authors:** Abdullah Mahmoud Riyahi

**Affiliations:** Department of Restorative Dental Science, Division of Endodontics, College of Dentistry, King Saud University, P.O. Box 60169, Riyadh 11545, Saudi Arabia

## Abstract

Three rooted lower first permanent molar represents one of the main anatomical variants which is a treatment challenge of clinicians. This study is aimed at presenting a case of a lower first molar with an additional root that was diagnosed and managed successfully using new techniques in endodontics. Tooth #46 was diagnosed as a necrotic pulp with symptomatic apical periodontitis. Different angle radiographs were obtained, and they clearly showed three roots. The procedure was completed under magnification and illumination using an operating microscope. The access cavity was modified to achieve straight line access for all the canals. Careful step-by-step instrumentation was performed using flexible NiTi rotary files. The canals were irrigated using 6% sodium hypochlorite. Afterwards, three-dimensional obturation was completed using warm vertical compaction. Knowledge of the anatomy and an early diagnosis are required to achieve high-quality root canal treatment.

## 1. Introduction

Complex root morphology and canal anatomy have been observed in the lower first molars [1–3]. The lower first molar usually has two roots, one located mesially and the other located distally. One major variant of this tooth morphology is the existence of a third root [4]. The additional root can be located distolingually (radix entomolaris) or mesiobuccally (radix paramolaris) [5, 6].

The occurrence of an additional root in the lower first molar varies across different populations. In the Chinese subpopulation, it was found to be 29% [7]. However, it was found to be as low as 0.68% in the German population [8].

The distolingual (DL) root is typically small and conical in shape with an apex that swings buccally [9]. This anatomical variation can present a clinical challenge during endodontic treatment [10]. Therefore, knowledge of this root morphology, the correct diagnosis, and proper management are important to achieve high-quality treatment.

Due to the importance of the subject, the literature has many case reports addressing the diagnosis and management of lower molar with an additional root. [11–13] With the current advances in endodontics, including operating microscopy, digital radiographs, cone beam computed tomography (CBCT), and heat-treated NiTi rotary file systems, such cases can be treated in a more predictable fashion.

This study aims to present a case of a lower first molar with a third root that was diagnosed and managed successfully using new techniques in endodontics.

## 2. Case

A 42-year-old male patient visited a dental clinic with the following chief complaint: “I have spontaneous pain in that tooth”. He pointed at tooth #46. The pain had started one month ago and was progressive. The patient had a noncontributory medical history with no known allergies. He had multiple restorations in the upper and lower arches with no history of LA complications.

An extraoral examination showed a symmetrical face, no head and neck lymphadenopathy, no TMJ clicking, and limited mouth opening. Tooth #46 responded negatively to the cold test and electric pulp test. However, there was tenderness upon percussion and palpation. The probing depth was within 3 mm, and there was no mobility in this tooth.

**Figure 1:**
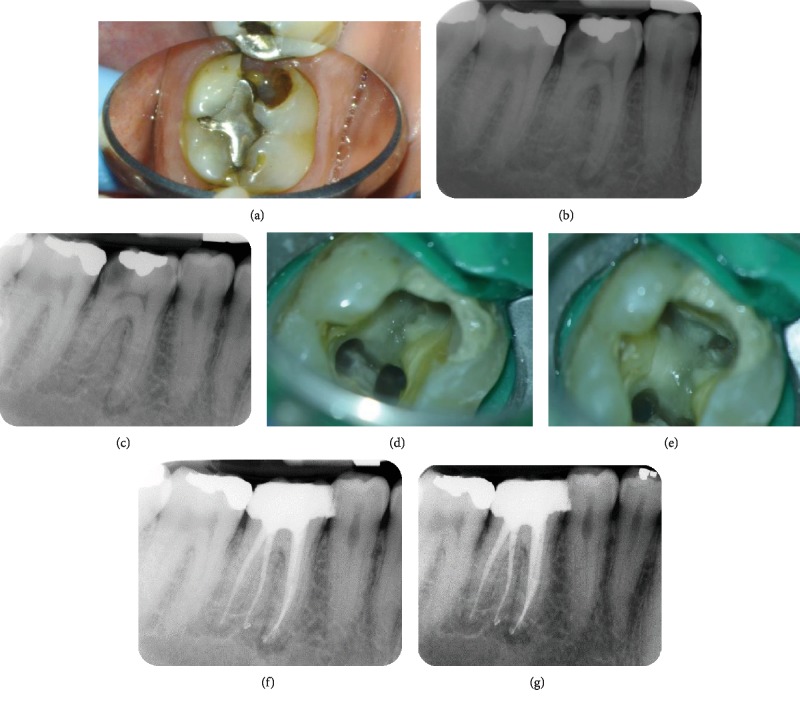
(a) Intraoral photo of tooth #46 showing a large carious lesion and defective amalgam restoration. (b, c) Periapical radiographs of different angles showing the additional root. (d, e) Access cavity preparation with four root canals located. (f, g) Final periapical radiographs from different angles.

Different angles of periapical radiographs for tooth #46 showed occlusal amalgam restoration, mesial and distal caries, periapical radiolucencies, and an additional root (Figure 1). The case was diagnosed as a necrotic pulp with symptomatic apical periodontitis.

Endodontic treatment was performed in two visits. In the first visit, under local anesthesia and rubber dam isolation, a modified access cavity was prepared. Four canals were located under operating microscope magnification. The apex locator was used to determine the working length. After the initial instrumentation was completed, calcium hydroxide was applied, and the access was closed with a cotton pellet and Cavit.

In the second visit, under local anesthesia and rubber dam isolation, the instrumentation was completed using ProTaper and Hyflex CM rotary file (Coltene Whaledent, Altstätten, Switzerland). The canals were irrigated using 6% sodium hypochlorite. Then, paper points were used to dry the canals. The canals were obturated using warm vertical compaction (Figure 1) with Gutta-percha and AH Plus sealer (Maillefer Dentsply, Ballaigues, Switzerland). Then, the access cavity was restored with Cavit. After two weeks, the clinical symptoms disappeared. The patient was referred for final restoration.

## 3. Discussion

Cases with complex canal anatomy, including lower molars with an additional root, require careful clinical and radiographic evaluations, magnification, and identification of all the canals through straight-line access, meticulous cleaning and shaping, copious irrigation, and three-dimensional obturation.

Since the permanent lower first molar can be three-rooted, the diagnosis of such morphology is essential at an early stage. This requires performing periodontal probing, which can enable the identification of the additional root. Furthermore, preoperative periapical radiographs obtained from different angles are needed to explore the existence of a third root [6].

The evaluation of case complexity is important before starting endodontic treatment. Once lower first molars with an additional root are identified, the difficulty of the case should be carefully assessed, and referral to an endodontist is usually required to manage such cases.

In general, different studies have evaluated finishing root canal treatment in a one visit or more. The clinicians can complete the treatment in one or multiple visits [14]. There is no evidence suggesting that it is better to complete the root canal treatment in a single visit or in multiple visits [15].

Due to the unusual location of the additional canal, the access cavity preparation should be modified accordingly to obtain straight-line access, which is necessary to enable proper chemomechanical instrumentation and three-dimensional obturation. Access modification has been reported for lower molars with additional roots [16].

Irrespective of the instrumentation technique to be used in these canals, initial canal negotiation is usually started with K-files of a small size. The small files made of stainless steel are distorted in a curve canal to estimate the curvature and anatomy present [10].

Irrigation is critical for the success of endodontic treatment. Sodium hypochlorite irrigant has a desired antimicrobial effect. Irrigation could be the only method to reach areas that are unreached through the mechanical action alone [17].

Furthermore, a severe curvature also requires special care to prevent instrument separation. Because of the possible presence of a severe canal curvature, the use of files that are more resistant to fracture by fatigue can be recommended. Therefore, heat-treated files can provide an expected advantage for cleaning and shaping such canals. Superior resistance to fracture by fatigue has been reported for heat-treated files [18].

Moreover, the magnification and illumination using an operating microscope are helpful for enhancing endodontic treatment quality. Furthermore, those features play an important role in difficult cases [19]. Modern endodontic treatment is more effective because of operating microscope usage [20]. In the management of lower molars with additional roots, the operating microscope is useful for locating the canals.

Finally, due to the difficulty of these cases, recent advances in endodontics should be utilized to facilitate high-quality root canal treatment.

## 4. Conclusion

Clinicians' knowledge about root canal anatomy variants in the lower first molars is of critical importance. The identification of lower molars with an additional root should start at an early stage. Proper clinical and radiographic examinations are required. Once the difficulty of the case is determined, referral to an endodontist may be the proper course of action for management.
